# The Relationship Between Osteoporosis and Intestinal Microbes in the Henan Province of China

**DOI:** 10.3389/fcell.2021.752990

**Published:** 2021-11-18

**Authors:** Qian Qin, Su Yan, Yang Yang, Jingfeng Chen, Hang Yan, Tiantian Li, Xinxin Gao, Youxiang Wang, Ang Li, Shoujun Wang, Suying Ding

**Affiliations:** ^1^ Health Management Center, The First Affiliated Hospital of Zhengzhou University, Zhengzhou, China; ^2^ College of Public Health, Zhengzhou University, Zhengzhou, China; ^3^ Gene Hospital of Henan Province, The First Affiliated Hospital of Zhengzhou University, Zhengzhou, China; ^4^ Department of Endocrinology, The First Affiliated Hospital of Zhengzhou University, Zhengzhou, China

**Keywords:** osteoporosis, gut microbiota, metagenomics, comparative genomics, metabolic pathway, phytate degradation

## Abstract

Osteoporosis (OP) is a chronic disease in the elderly, and China is entering an aging demographic trend. In recent years, increasing evidence has demonstrated that probiotics can treat osteoporosis. This study aimed to explore the relevant mechanisms and to validate the beneficial effect on osteoporosis by high-throughput metagenome-wide gene sequencing in humans. In this study, compared with controls, several species had altered abundances, and specific functional pathways were found in the OP group. At the species level, the species that had increased in OP individuals were positively correlated to bone resorption markers and negatively correlated to 25-OH-D3 and bone formation markers, with *Streptococcus sanguinis* showing the strongest relevance, followed by *Streptococcus gordonii*, *Actinomyces odontolyticus*, and *Olsenella unclassified.* Additionally, *Actinomyces graevenitzii*, enriched in the OP group, was positively correlated to inflammation indicators that included white blood cell (WBC), neutrophil count (NEC), and the neutrophil-to-lymphocyte ratio (NLR) (*p* < 0.05). Conversely, the levels of *Akkermansia muciniphila*, *Bacteroides eggerthii*, *Bacteroides fragilis*, *Bacteroides uniformis*, and *Butyricimonas synergistic* were increased in the control group, which had a negative correlation with bone resorption markers and positive correlation with bone formation markers and 25-OH-D3. Additionally, *Bacteroides fragilis* had a negative correlation with inflammation indicators (WBC, NEC, and NLR) and the above pathways (*p* < 0.05). Functional prediction revealed that 106 metabolic pathways, enriched in the OP group, were significantly higher than in the control group (*p* < 0.05). In particular, pathways related to LPS biosynthesis, phytate degradation, lactate acid, and ethanol fermentation were more abundant in the OP group than in the control and were positively related to WBC and NEC. Taken together, several species with altered abundances and specific functional pathways were found in OP individuals. The role of phytases in OP provides novel epidemiological evidence to elucidate the underlying microbiota-relevant mechanisms in bone mineralization and should be explored further.

## Introduction

With changes in living conditions and an increased elderly population, osteoporosis has become a common chronic disease. Particularly in elderly patients, osteoporosis fractures cause substantial morbidity and mortality (Hofbauer and Rachner, 2015). In China, it is estimated that the prevalence of osteoporosis is approximately 13%, and the elderly demographic trend may incur an annual cost due to osteoporotic fractures from $27.48 billion in 2020 to $581.97 billion in 2050 (Si et al., 2015). Osteoporosis and osteoporotic fractures affect quality of life and cause a huge economic burden on families and society. To prevent and treat osteoporosis in addition to improving lifestyle (Sun exposure, strengthening exercise, and nutritional supplements), the main measure is drug treatment. However, drug treatments, which include calcium, vitamin D, diphosphates, calcitonin, hormone replacement therapy, such as estradiol, androgen, and testosterone, and selective estrogen receptor modulators, have indications and adverse effects. Consequently, early prediction and new treatment methods for osteoporosis must be urgently identified to minimize the effect of fractures.

Recent evidence suggests that gut microbiota is closely related to mineral absorption and osteoporosis. Compared with age-matched mice with healthy microbiota, bone morphology is altered in bacteria-free mice (Blanton et al., 2016). *Lactobacilli* strain supplementation maintains normal bone growth of infant mice and *Bifidobacterium* increases bone mineral density in rats (Rodrigues et al., 2012; Schwarzer et al., 2016). This is consistent with the femoral neck bone density in elderly men, which increased by 5% after consuming kefir fermented milk (Han et al., 2017). However, another study observed no significant change in BMD in postmenopausal women with supplemented GeriLact probiotics, but reduced serum levels of parathyroid hormone and tumor necrosis factor-α (TNF-α) (Jafarnejad et al., 2017). Some pathophysiological mechanisms have been proposed to explain these interesting phenomena. Short-chain fatty acids (SCFAs), which are metabolites of intestinal microorganisms, directly absorb calcium via the paracellular pathway by increasing the villi structure, and surface area of the small intestines. SCFAs also reduce the pH in the intestines, which increases mineral solubility and enables calcium to be absorbed more easily (McCabe et al., 2015). Probiotics increase osteoprotegerin levels, thereby reducing osteoclast production by affecting the immune system and proinflammatory cytokines such as TNF-α and interleukin-1 (IL-1) (Ohlsson et al., 2014).

Most studies have observed changes in bone mass after supplementation with lactic acid bacteria. Some reports have investigated intestinal microorganisms in osteoporotic populations of Henan Province in China. This study aimed for early prevention and provides a new therapeutic direction for osteoporosis by analyzing the diversity of intestinal microorganisms and metabolites between osteoporotic and normal bone masses in Henan Province.

## Materials and Methods

### Study Participants

**FIGURE 1:**
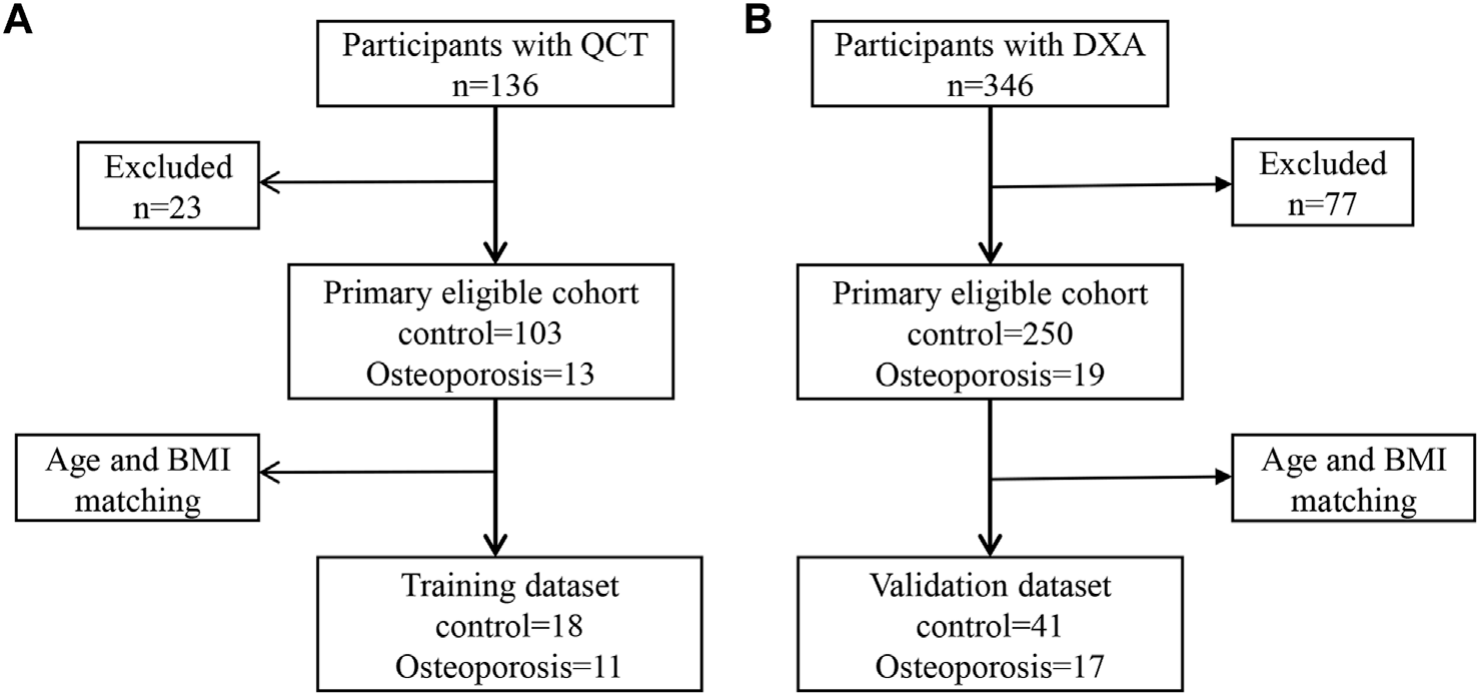
Metagenomic genome-wide association analysis of the primary cohort. **(A)** Analysis of the primary cohort of osteoporosis measured by quantitative computed tomography (QCT). **(B)** Analysis of the primary cohort of osteoporosis measured by dual-energy x-ray absorptiometry (DXA).

Study participants aged 55–75 years were recruited at our hospital from January 2018 to July 2019. BMD assessment was performed by quantitative computed tomography (QCT) for 136 participants or dual-energy x-ray absorptiometry (DXA) for 346 participants. The inclusion criterion was health checkups over 55–75 years of age. Exclusion criteria were as follows: 1) patients with metal implants within the upper abdomen scan; 2) alcohol abuse or smokers, history of surgery within 1 year, chronic diseases such as hypertension, diabetes, coronary heart disease, or tumors; 3) diseases and drug treatments that affect BMD and antibiotic use; and 4) participants who have had previous fracture history (accidental fracture and fragility fractures). Finally, to construct a simple noninvasive model of OP, 29 participants with QCT were included in the training dataset (Figure 1A), while 58 participants with DXA were assigned to the validation dataset (Figure 1B). All experiments that involved humans were reviewed and approved by the Ethics Committee of the First Affiliated Hospital of Zhengzhou University (2018-KY-56). All participants provided informed consent.

### Data Collection

A questionnaire and a physical examination were conducted by trained staff at the First Affiliated Hospital of Zhengzhou University to collect data on demographic characteristics, medical history, behavioral risk factors, height, weight, waist circumstance (WC), and blood pressure. Participants were required to have blood drawn after 12 h of fasting. Serum test results [white blood cell count (WBC), neutrophil count (NEC), high-density lipoprotein cholesterol (HDL), low-density lipoprotein cholesterol (LDL), triglycerides (TGs), total cholesterol (TC), fasting blood glucose (FBG), glycated hemoglobin A1c, serum uric acid, parathyroid hormone (PTH), type 1 procollagen N-terminal propeptide (PINP), β-crosslaps (β-CTX), and 25-hydroxy vitamin D3 (25-OH-D3)] of the participants were obtained from hospital laboratories. Additionally, bioelectrical impedance analysis (BIA) (InBody 770, InBody Co., Ltd., Korea) was used to analyze the body composition that included the lean body mass, fat-free mass (FFM), FFM of the trunk, and FFM of the arms and legs. The BMD, T-score, and Z-score were measured at the T11–L4 level by CT scans (Brilliance iCT Elite FHD device) with scan parameters of 120 kV, 41 mAs for weight >70 kg, 19 mAs for weight ≤70 kg, 5-mm slice thickness and spacing for scanning, 1-mm slice thickness and spacing for reorganization, 0.5-s rotation time, 5,122-pixel matrix, and 500-mm display field of view and bed height based on the midaxillary line, which were automatically calculated by using QCT PRO V6.1 software (Mindways, United States). The specific BMD measured by QCT is shown in Supplementary Figure S1. Additionally, the T-score and Z-score were measured by DXA (Lunar iDXA, United States). The specific BMD measured by DXA is shown in Supplementary Figure S2.

All CT examinations were performed by two experienced radiologists. On the same day, fecal samples were collected and stored at −80°C until analysis.

### Diagnostic Criteria of Osteoporosis

Diagnostic criteria referred to the International Society for Clinical Densitometry on the QCT BMD measurement operation guide (Camacho et al., 2020). The diagnostic criteria are as follows: <80 mg/cm^3^ for OP and >120 mg/cm^3^ for normal.

Diagnostic criteria referred to “normal, osteopenia, and osteoporosis” as recommended by a WHO working group (Dimai, 2017). The diagnostic criteria were a T-score of ≤ −2.5 for OP and a T-score of ≥ −1.0 for normal.

### Microbiome Composition and Function Profiling

DNA was extracted from 88 stool samples using a MagPure Stool DNA KF kit in accordance with the instructions of the manufacturer. DNA library construction based on DNA nanospheres and shotgun metagenomic sequencing based on combined probe anchoring synthesis were performed on all samples (MGI 2000, MGI, Shenzhen, China). The overall accuracy (≥0.8) control strategy described above was used to carry out quality control on original sequenced reads to filter out low-quality reads.

Metagenomic classification of sequenced libraries was carried out through MetaPhlAn2 to obtain standard relative abundance values of species at all levels. First, there was a comparison between the sequence and marker. The MetaPhlAn2 classifier finally compared the metagenomic reads against a precomputed marker catalog using nucleotide BLAST searches to provide clade abundances for one or more sequenced metagenomes. This was followed by content calculation. The classifier normalized the total number of reads in each clade by the nucleotide length of its markers and provided the relative abundance of each taxonomic unit while taking into account any markers specific to subclades. The microbial clade anomaly was, thus, estimated by normalizing read-based counts to the average genome size of each clade. Finally, a map of the gut microbes, which included bacteria, archaea, eukaryotes, and viruses, was constructed. Community functional profiles of gut microorganisms were further generated using HumanN2 (HMP Unified Metabolic Analysis Network 2).

### Statistical Analysis

Statistical analyses were performed using R program version 4.0.2. Standardized statistical test methods were used to analyze the results of demography and laboratory tests. Categorical variables are represented by counts. Fisher’s tests were used for association testing. Continuous variables are expressed as means ± SD. Analysis of differences between groups was performed by normality and homogeneity tests. A value of *p* ≥ 0.05 was considered normal and homogeneous, followed by Student’s t-test or Wilcox test. A value of *p* < 0.05 was considered statistically significant. We used R package “vegan” to calculate the Shannon and Gini alpha diversity index and Hellinger, Bray, JSD, and Pearson beta diversity index of each sample. Principal coordinate analysis was performed by the R program “ade4” for visual analysis. The Wilcox test was used to analyze differences between groups of microbiota and pathways. A value of *p* < 0.05 was considered statistically significant. Spearman correlation analysis was used to assess the correlation between differential flora and covariates. The “corrplot” package was used for visualization. Finally, in accordance with the results of difference analysis, we used logistic regression to establish a prediction model. The samples were divided into training and test sets. A logistic regression model was established in the training set, and verification was carried out in the substitution test set. A ROC curve was used to compare the effectiveness of prediction models.

## Results

### Clinical Characteristics of Participants

In total, 88 participants were enrolled to analyze stool samples and obtain clinical information. Thirty participants were diagnosed with OP, and 59 participants with a normal BMD were the control group. Twenty-eight participants (11:18) were included in the training dataset, while 60 participants (19:41) were assigned to the validation dataset. Compared with the control group, there were no significant differences in sex, age, or biochemical parameters of metabolic syndrome in the OP group of the training dataset, which indicated that the influence of intestinal microbes caused by a common reason, chronic diseases, or nutritional factors could be excluded. No significant difference in PTH was found between the two groups. Moreover, PTH in the OP group within the normal range ruled out secondary PTH-induced osteoporosis. OP individuals had a lower BMD, bone formation markers (PINP), lean body mass, and 25-OH-D3 than the control group (<0.05; Table 1). However, bone resorption markers (β-CTX), NEC, and NLR in the OP group were significantly (*p* < 0.05; Table 1) higher than those in the control group.

**TABLE 1:** BMD, body composition, and clinical information of OP and control groups.

	Control	OP	*p*
age	58.72 ± 4.06	62.00 ± 7.33	0.131
Sex (female/male)	4/14	5/6	0.237
BMD (mg/cm^3^)	150.72 ± 26.62	58.08 ± 13.00	<0.001*
WC (cm)	93.33 ± 6.04	85.86 ± 11.70	0.187
BMI (kg/m^2^)	25.28 ± 2.53	23.94 ± 3.03	0.212
Lean body mass (kg)	52.02 ± 3.29	41.63 ± 7.13	<0.001*
FFM (kg)	55.13 ± 3.44	44.14 ± 7.48	<0.001*
FFM of trunk (kg)	24.81 ± 1.18	20.04 ± 3.60	<0.001*
FFM of arms and legs (kg)	23.30 ± 1.54	18.46 ± 3.70	<0.001*
SBP (mmHg)	129.76 ± 15.83	136.73 ± 27.02	0.396
DBP (mmHg)	79.06 ± 10.48	78.27 ± 13.03	0.864
FBG (mmol/L)	5.89 ± 1.75	5.38 ± 0.49	0.36
HbA1c (%)	6.25 ± 1.04	5.73 ± 0.28	0.136
TC (mmol/L)	4.75 ± 0.71	4.93 ± 1.28	0.628
TG (mmol/L)	1.47 ± 0.74	1.31 ± 0.63	0.552
HDL (mmol/L)	1.29 ± 0.25	1.41 ± 0.29	0.223
LDL (mmol/L)	2.93 ± 0.64	3.11 ± 0.92	0.554
SUA (mmol/L)	327.17 ± 54.60	294.27 ± 62.28	0.147
WBC (10^9^/L)	5.19 ± 0.87	5.98 ± 1.97	0.148
NEC (10^9^/L)	2.87 ± 0.61	3.56 ± 1.18	0.046*
NLR (%)	1.64 ± 0.41	2.11 ± 0.73	0.037*
PTH (pg/ml)	37.26 ± 8.65	38.70 ± 11.47	0.646
PINP (ng/ml)	34.29 ± 5.86	29.09 ± 7.19	0.046*
β-CTX (ng/ml)	0.21 ± 0.06	0.46 ± 0.26	0.004*
25-OH-D3 (ng/ml)	29.55 ± 9.99	18.54 ± 5.31	<0.001*

WC, waist circumference; BMI, body mass index; BMD, bone mass density; FFM, fat-free mass; SBP, systolic blood pressure; DBP, diastolic blood pressure; FBG, fasting blood glucose; HbA1c, glycated hemoglobin A1c; TC, total cholesterol; TG, triglyceride; HDL, high-density lipoprotein; LDL, low-density lipoprotein; SUA, serum uric acid; WBC, white blood cell; NEC, neutrophil count; NLR, neutrophil to lymphocyte ratio; PTH, parathyroid hormone; PINP, type 1 procollagen N-terminal propeptide; 25-OH-D3, 25-hydroxy vitamin D3; OP, osteoporosis. Statistical significance was defined as *p* < 0.05. *p < 0.05.

### Analysis of Intestinal Flora Diversity

**FIGURE 2:**
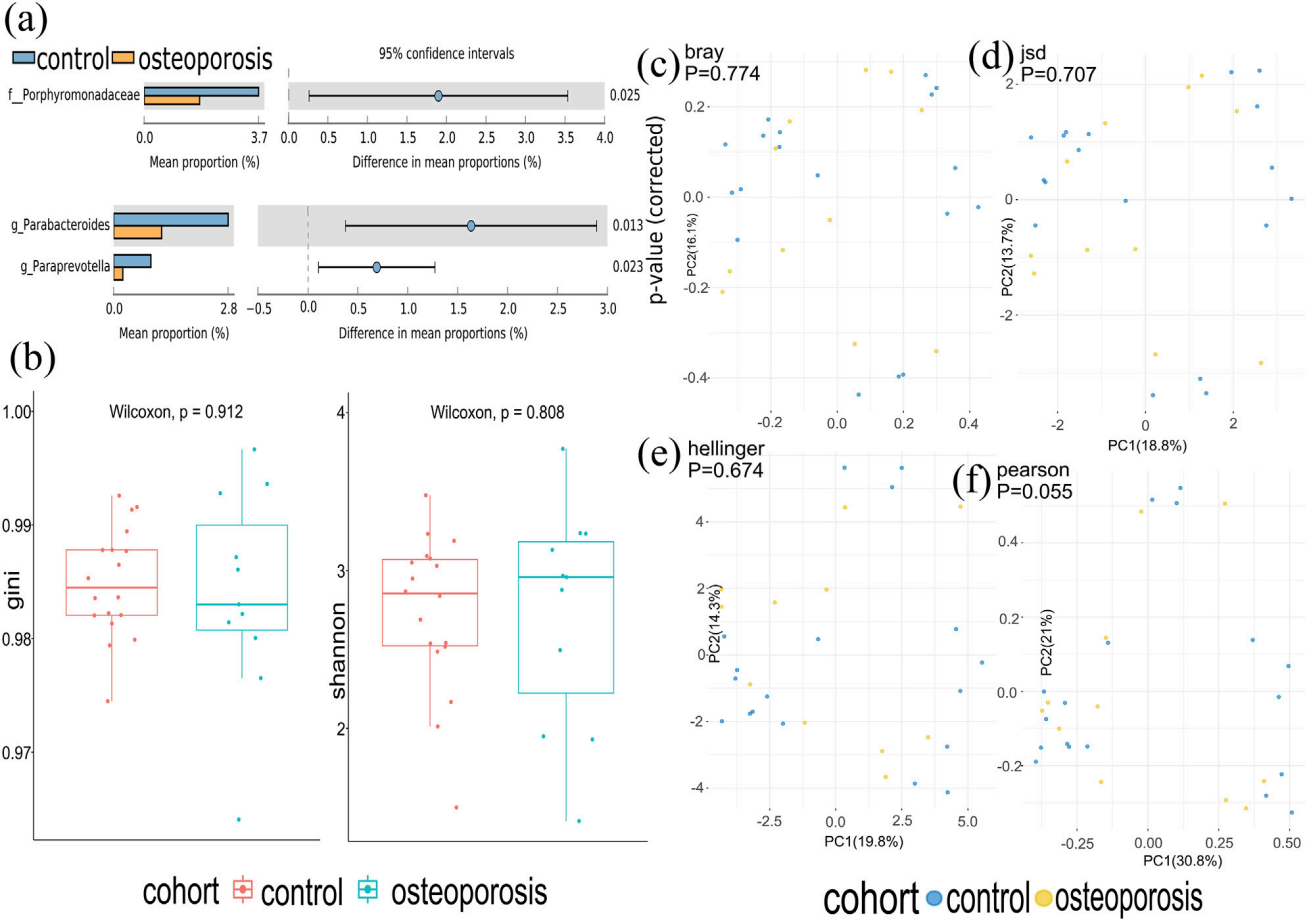
Gut microbiota composition of osteoporosis (OP) and control groups. **(A)** Compared with the control group, relative abundances of Porphyromonadaceae, Parabacterodies, and Parapreotella were decreased in the OP group (*p* < 0.05). There were no significant differences in the phyla of the OP group compared with the control group (*p* > 0.05). **(B)** Alpha diversity was measured by both Shannon and Gini indices for comparisons between OP and control groups. **(C–F)** Beta diversity between OP and control groups. Beta diversity was calculated by Hellinger, Jensen–Shannon divergence (JSD), Bray, and Spearmen distances. No significant differences were found between the OP and control groups.

Metagenomics sequences were analyzed in 29 fecal samples to categorize sequences into phyla and genera based on their closest match in the reference database. We used STAMP to analyze the different microbiota at all levels (phylum to genus) and found that one family and two genera (*p* < 0.05; Figure 2A) were different between OP and control groups. No significant difference was observed at phyla and species levels.

At the species level, there was a very similar level of microbiome diversity in the OP group with the alpha diversity compared with that in the control group as measured by both Shannon and Gini indexes (*p* = 0.808 and *p* = 0.912) (Figure 2B). To compare the species-level beta diversity of OP and control groups, the Hellinger, Jensen–Shannon divergence (JSD), Bray, and Spearmen distances were calculated. This analysis revealed no significant difference in the species-level beta diversity between OP and control groups (*p* = 0.674, *p* = 0.707, *p* = 0.774, and *p* = 0.055, respectively, Figures 2A–F).

### Analysis of Microbiota Composition and Associations Among Gut Microbiomes and Clinical Characteristics

#### Analysis of the Microbiota Composition

**FIGURE 3:**
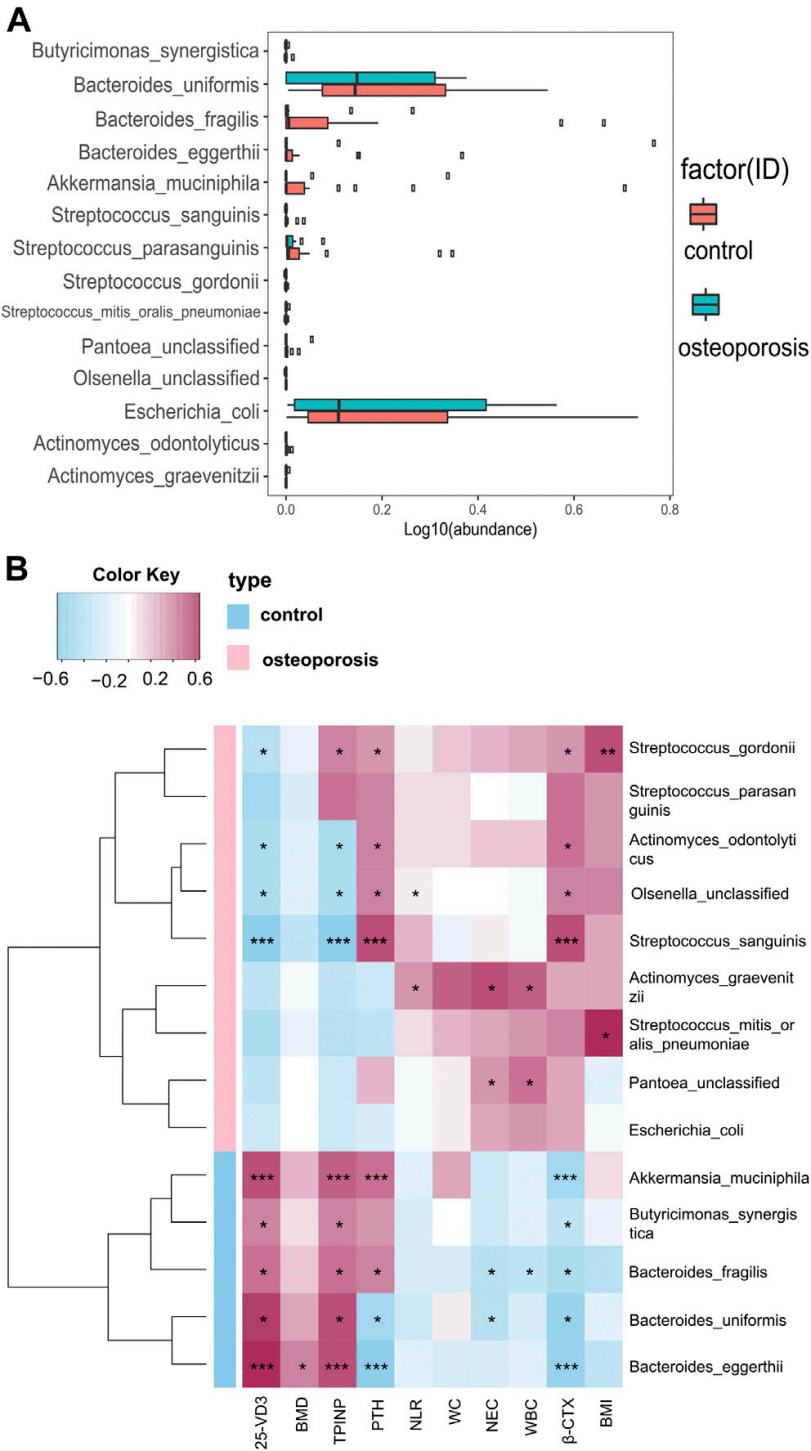
Relative abundances of bacterial species between OP and control groups, and relationships between gut microbiota abundances and clinical indicators. **(A)** Wilcoxon tests were used to analyze the relative abundances of bacterial species, which showed significant differences in 14 species with five enriched in the control group and nine enriched in the OP group (*p* < 0.05). **(B)** Correlation matrix for species and clinical parameters. Yellow and red signify a positive correlation, while blue signifies a negative correlation. Cells with an asterisk indicate *p* < 0.05.

Compared with the control group, we identified 20 species with significantly different abundances in the OP group. After removing species with low occurrence and abundance, 14 species with significantly different abundances remained (*p* < 0.05; Figure 3A), which indicated an altered composition of the gut microbiota in the OP group. In particular, the levels of *Akkermansia muciniphila*, *Bacteroides eggerthii*, *Bacteroides fragilis*, *Bacteroides uniformis*, and *Butyricimonas synergistic* were decreased, and the abundances of *Actinomyces graevenitzii*, *Actinomyces sodontolyticus*, *Olsenella unclassified*, *Pantoea unclassified*, *Streptococcus gordonii*, *Streptococcus mitis oralis pneumoniae*, *Streptococcus parasanguinis*, *Streptococcus sanguinis*, and *pathogenic bacteria*, such as *Escherichia coli*, were increased in the OP group (*p* < 0.05; Figure 3A).

#### Correlations Between Gut Microbiota Abundances and Bone Mass Density Measurements or Inflammation Levels and Bone Metabolism Markers

We used Spearman’s correlation analysis to explore correlations between species abundances and clinical characteristics. We found that *Bacteroides eggerthii* was enriched in the control group with the strongest positive correlation with BMD (*r* = 0.378, *p* < 0.05; Figure 3B). Furthermore, we analyzed the correlation between gut microbiota and inflammation levels or bone metabolism markers. All five species enriched in the control group had a positive correlation with the bone formation marker (PINP) and 25-OH-D and had a negative correlation with the bone resorption marker (β-CTX), with *Akkermansia muciniphila* and *Bacteroides eggerthii* showing the strongest relevance, followed by *Butyricimonas synergistic*, *Bacteroides fragilis*, and *Bacteroides uniformis*. Moreover, all five species enriched in the control group had a negative correlation with WBC, NEC, and NLR, with *Bacteroides fragilis* showing the strongest correlation with WBC and NEC (*r* = −0.313 and −0.348, *p* < 0.05; Figure 3B).

The four species enriched in the OP group had a positive correlation with β-CTX and a negative correlation with PINP and 25-OH-D3, with *Streptococcus sanguinis* showing the strongest relevance, followed by *Streptococcus gordonii*, *Actinomyces odontolyticus*, and *Olsenella unclassified*. *Actinomyces graevenitzii*, enriched in the OP group, had the strongest positive correlation with WBC, NEC, and NLR (*r* = 0.435, 0.532, and 0.305, *p* < 0.05; Figure 3B). All specific correlations are shown in Supplementary Table S1.

### Functional Shifts in the Microbiome Characteristics of Different Participants and Relationships Between Functional Shifts and Bone Mass Density or Clinical Characteristics

#### Analysis of Functional Shifts in Microbiome Characteristics

**FIGURE 4:**
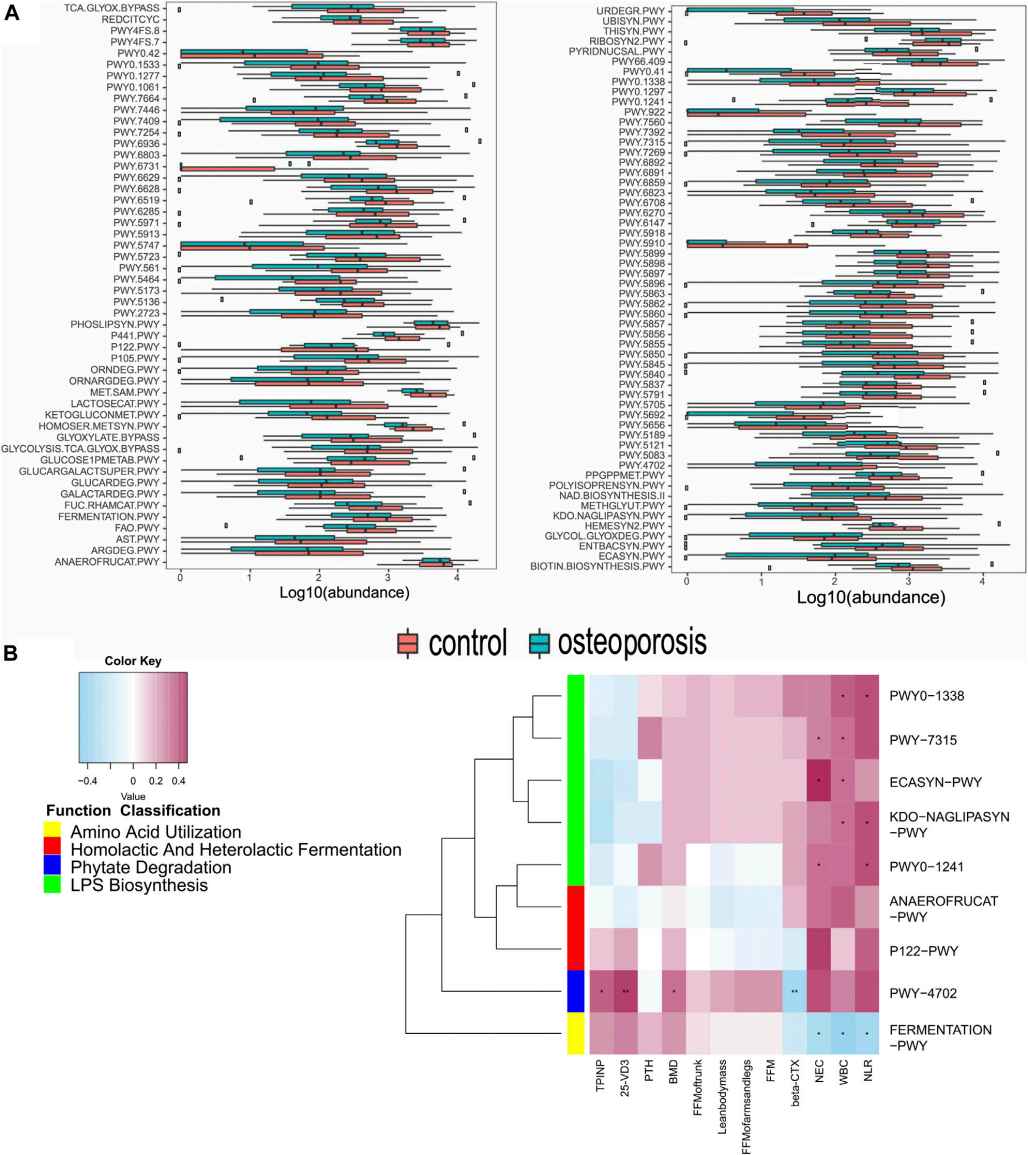
Functional shifts in bacterial species between OP and control groups, and relationships between functional shifts and BMD or inflammation levels. **(A)** Functional shifts in bacterial species. MetaCyc pathways with different abundances between healthy controls and the OP groups (*p* < 0.05, Wilcoxon rank sum test). **(B)** Spearman’s correlation matrix for OP-correlated pathways and BMD measurements or inflammation levels. Blue signifies a negative correlation, while yellow and red signify a positive correlation. Cells with an asterisk indicate *p* < 0.05.

We constructed functional profiles for each sample using microbial MetaCyc pathways. After removing low abundance pathways, 106 MetaCyc pathways, enriched in the OP group, were found to be significantly higher than in control groups (*p* < 0.05; Figure 4A). Approximately 44.3% of the pathways relevant to glycan degradation, lipid metabolism, the TCA cycle, and amino acid biosynthesis or utilization had more abundance in the OP group than in the control group. Among them, 47.1% of the pathways enriched in OP participants were responsible for cofactor, carrier, vitamin and siderophore, and metallophore biosynthesis (these pathways contribute largely to redox reactions and the TCA cycle). Importantly, three pathways were responsible for mixed acid, homolactic, and heterolactic fermentation (precursor metabolite fermentation is a process by which sugars, such as glucose, fructose, and sucrose, are converted to cellular energy and metabolic byproducts lactate acid and ethanol). PWY-4702 was responsible for phytate degradation that releases phosphates. Five pathways (PWY0-1338, PWY0-1241, PWY-7315, KDO-NAGLIPASYN-PWY, and ECASYN-PWY) were responsible for lipopolysaccharide (LPS) biosynthesis.

#### Correlations Between Functional Shifts of the Microbiome and Bone Mass Measurements or Inflammation Levels and Bone Metabolism Markers

Most of these pathways between the two groups were responsible for the metabolism of glycolipids and amino acids. Therefore, we focused on correlations between the clinical indicators and functional pathways responsible for inflammation, short-chain fatty acids, and phytase. A total of 104 OP-enriched pathways (except for PWY-922, FERMENTATION-PWY, and PWY-7392) were positively related to WBC and NEC (Figure 4B). In particular, these pathways included those responsible for LPS biosynthesis (KDO-NAGLIPASYN-PWY, ECASYN-PWY, PWY0-1338, PWY0-1241, and PWY-7315). Conversely, FERMENTATION-PWY was negatively related to inflammation indicators (WBC and NEC). Additionally, PWY-4702, responsible for phytate degradation, was positively related to BMD, 25-OH-D3, and the bone formation marker (PINP), but was negatively related to the β-CTX (*r* = 0.0.311, 0.419, 0.337, and −0.357, *p* < 0.05; Figure 4B).

### Relationship Between Functional Shifts and Microbiome Characteristics

**FIGURE 5:**
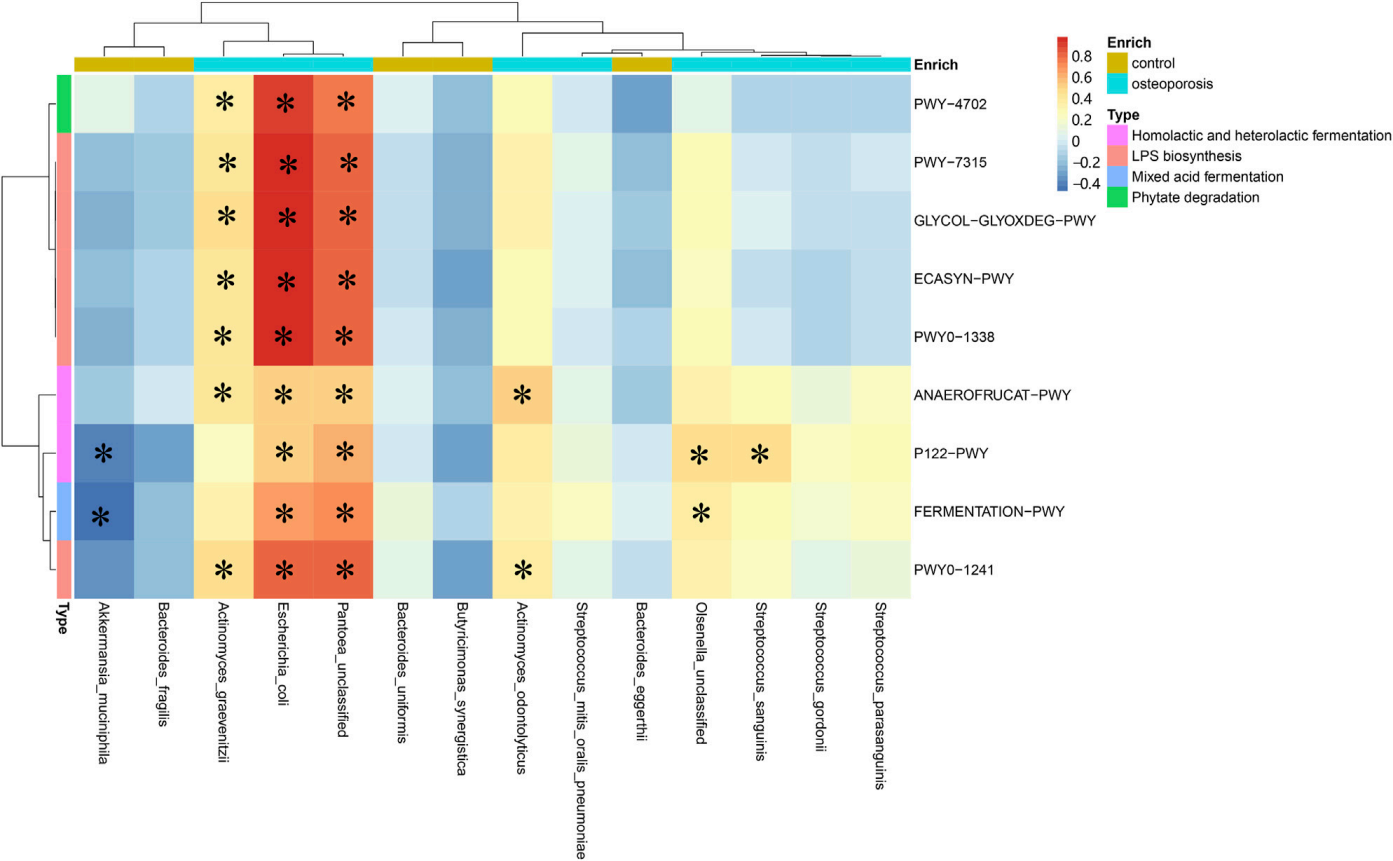
Spearman’s correlation matrix for microbial pathways and species. Blue signifies a negative correlation, while yellow and red signify a positive correlation. Cells with an asterisk indicate *p* < 0.05.

Spearman’s correlation analysis was used to explore the relationships between species abundances and functional shifts. The pathways responsible for phytate degradation, LPS biosynthesis, and lactate acid and ethanol fermentation were positively related to *Actinomyces graevenitzii*, *Actinomyces odontolyticus*, *Escherichia coli*, and *Pantoea unclassified* enriched in the OP group (*p* < 0.05; Figure 5). All specific correlations are shown in Supplementary Table S2.

### Establishment and Verification of a Predictive Model of Osteoporosis by the Gut Microbiome

**FIGURE 6:**
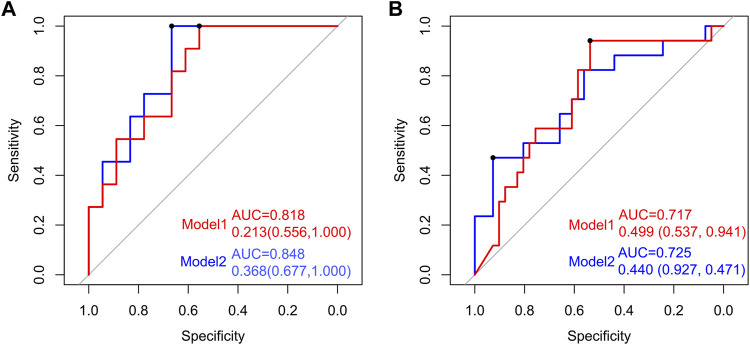
Establishment and verification of the predictive model of OP by the gut microbiome. Receiver operating characteristic (ROC) curves for predicting OP in the training and test datasets. The red line indicates the ROC of model 1 (*Streptococcus sanguinis*, *Akkermansia muciniphila*, and *Bacteroides eggerthii*) based on multifactor logistic regression. The blue line signifies the ROC of model 2 (*Actinomyces graevenitzi*i, *Actinomyces odontolyticus*, *Streptococcus sanguinis*, *Akkermansia muciniphila*, *Bacteroides eggerthii*, and *Butyricimonas synergistica*).

To establish a simple noninvasive model of OP, 28 participants (11:18) were included in a training dataset, while 60 participants (17:41) were assigned to the test dataset. A multifactor logistic regression method was further used to establish the prediction model, which showed that three species (*Streptococcus sanguinis*, *Akkermansia muciniphila*, and *Bacteroides eggerthii*) were factors of OP in the training dataset (Figure 6). The area under receiver operating curve (AUC) of model 1 was 0.818 and 0.717 in the training and test datasets, respectively. Additionally, by analyzing the correlation between the gut microbiome and clinical characteristics, operational model 2 was constructed, which included six species (*Actinomyces graevenitzii*, *Actinomyces odontolyticus*, *Streptococcus sanguinis*, *Akkermansia muciniphila*, *Bacteroides eggerthii*, and *Butyricimonas synergistica*). The AUC of model 2 was 0.848 and 0.725 in the training and test datasets, respectively, which was higher than those of model 1 (Figure 6).

## Discussion

This study investigated specific changes in the gut microbiota composition and functional relationship in a human OP cohort in Henan province of China by metagenomic sequencing. To date, the underlying mechanisms by which gut microbes affect BMD remain unclear. However, we found underlying and interesting relationships between the composition and function of gut microbes and OP. At the species level, *Actinomyces graevenitzii*, *Actinomyces sodontolyticus*, *Olsenella unclassified*, *Pantoea unclassified*, *Streptococcus gordonii*, *Streptococcus mitis oralis pneumoniae*, *Streptococcus parasanguinis*, and *Streptococcus sanguinis* were enriched in the OP group, and *Akkermansia muciniphila*, *Bacteroides eggerthii*, *Bacteroides fragilis*, *Bacteroides uniformis*, and *Butyricimonas synergistic* were enriched in the control group, which had a negative correlation with inflammatory parameters with *Bacteroides eggerthii*, showing the strongest positive correlation with BMD. Conversely, the species enriched in OP was positively related to the bone resorption markers and negatively related to 25-OH-D3 and bone formation markers. Furthermore, LPS biosynthesis pathways were positively related to inflammatory parameters and the species enriched in the OP group and negatively related to the species enriched in the control group, which implied that it is involved in bone metabolism.

In particular, *Streptococcus sanguinis* and *Actinomyces odontolyticus*, enriched in the OP group, had a positive correlation with bone resorption marker (β-CTX) and a negative correlation with bone formation markers PINP and 25-OH-D3. *Actinomyces* in the OP group induces bone invasion in bisphosphonate-related OP of the jaw, and antimicrobial therapy may lead to better clinical outcomes (Arranz Caso et al., 2012), which indicates that *Actinomyces* may contribute to inflammation that causes subsequent bone necrosis (Schipmann et al., 2013; Das et al., 2019). Additionally, *Streptococcus sanguinis* has a bone-resorptive activity by increasing osteoclast differentiation and reducing osteoblasts with inhibition of Runx2 expression (Park et al., 2019). This is consistent with the results of our study, which showed a higher level of inflammation in the OP group, and the species enriched in the OP group were positively correlated to WBC, NEC, and NLR, especially *Actinomyces graevenitzii*. Therefore, we considered that the bacteria enriched in the OP group caused OP by mediating inflammation. Furthermore, functional genetic analysis showed that the number of pathways responsible for the TCA cycle, metabolism of glycans, lipid, and amino acid, and redox reactions were more abundant in the OP group than in the control group. In particular, the pathways of LPS biosynthesis were positively related to NEC and *Actinomyces graevenitzii*, *Actinomyces odontolyticus*, *Escherichia coli*, and *Pantoea unclassified*, enriched in the OP group, which suggests that the gut microbiota plays a role in causing low-grade inflammation. LPS induces bone loss by affecting osteoclast proliferation, downregulating osteoprotegerin, and upregulating osteoclastic cytokines due to damaged integrity of the intestinal mucosa and increased gut permeability, which indirectly induce an inflammatory response with increased levels of TNF-α, IL-1, and INF-γ (Sjogren et al., 2012; Kitaura et al., 2020; Rettedal et al., 2021). Thus, we inferred that overproduction of LPS by the gut microbiota contributes to bone mineral loss, possibly via inflammation-related pathways.

*Akkermansia muciniphila* restores bone destruction and improves revascularization of bone fractures by reducing gut permeability and inhibiting systemic inflammatory cell infiltration mediated by increasing anti-inflammatory IL-10 and decreasing anti-inflammatory IL-12 (Huck et al., 2020; Liu et al., 2020). Additionally, *Akkermansia muciniphila* increases expression of junctional integrity markers such as integrin β1, E-cadherin, and ZO-1 in TIGK cells (Huck et al., 2020). Consistent with our results, we observed that the levels of *Akkermansia muciniphila and Bacteroides eggerthii* were decreased in the OP group and were negatively related to bone resorption marker β-CTX and positively related to bone formation markers PINP and 25-OH-D3. Additionally, *Bacteroides eggerthii*, which was positively correlated to BMD, enhances innate and adaptive immunities and alleviates allergen-induced airway inflammation in mouse offspring (Kao et al., 2020). As the course of chronic kidney disease progresses, the level of *Bacteroides eggerthii* decreases gradually, which implies that *Bacteroides eggerthii* is of particular value for early diagnosis. Thus, we speculated that *Bacteroides eggerthii* causes OP by participating in the synthesis of 25-OH-D3 in the kidneys (Wu et al., 2020). Notably, the level of *Butyricimonas synergistic* was decreased in the OP group. *Butyricimonas synergistica*, a butyric acid/SCFA-producing bacteria, reduces LPS production, which implies that increasing *Butyricimonas synergistica* by EPA/DHA supplementation attenuates adipose inflammation by upregulating glucose transporter 4 and Akt phosphorylation, indicating an improvement in insulin signaling (Zhuang et al., 2020). Furthermore, functional gene analysis showed that pathways enriched in the OP group were related to lactate fermentation that reduces the level of SCFAs. The inflammation system is critical for normal bone remodeling and initiation of bone loss (Harris et al., 2009). Thus, we speculated that the reduction in SCFA-producing bacteria promoted the proliferation of LPS-producing bacteria, which increases intestinal permeability and leads to chronic low-grade systemic inflammation. SCFAs inhibit Wnt10b signaling in mesenchymal precursor cells, which stimulates osteoblast production and inhibits adipocyte production (Arpaia et al., 2013; Weaver, 2015; Hernandez et al., 2016).

Interestingly, PWY-4702 is responsible for phytate degradation. Phytate is the most abundant myoinositol phosphate. The potential of phytate to form very stable complexes with minerals and proteins confers this molecule with its notorious antinutritional properties, which indicates its ability to chelate mineral cations to compromise mineral absorption (Belgaroui et al., 2014; Humer et al., 2015). Degradation of phytate is achieved by a group of enzymes called phytases that are capable of initiating stepwise release of phosphate from phytate. Ultimately, phytases enhance utilization of phosphate and improve the nutritional value of plant-based foods by increasing the availability of minerals chelated in phytin. However, humans and monogastric animals lack hydrolytic phytases in their digestive tract. This is consistent with our study that revealed enrichment of PWY-4702 in the OP group. To respond to compensatory bone metabolism in OP patients, the pathway responsible for the release of phosphate is increased by gut microbes. This pathway was positively correlated to bone formation indicators and negatively related to bone resorption indicators, which further indicates that this pathway, involved in the species, may compensate for bone formation. Notably, a previous study showed that phosphorus digestibility and bone mineralization are improved, and fecal phosphate excretion is reduced, by microbial phytase supplementation in pig feed (Harper et al., 1997; Oster et al., 2016). Thus, bone resorption may be promoted by phytase-producing bacteria.

## Conclusion

This study had several limitations. The sample size was small, and the BMD of the verification dataset was measured by DXA. We also did not collect some lifestyle information, such as sunlight exposure and exercise methods. Nevertheless, this study found extensive and interesting changes in association with the gut microbiota composition and functions with OP in Chinese patients, which were characterized by increased levels of *Actinomyces* and *Streptococcus*, decreased levels of *Akkermansia*, *Bacteroides*, and *Butyricimonas synergistic*, and alteration of several functional pathways. In particular, bone resorption may be inhibited and bone formation promoted by phytase-producing bacteria. Additionally, the higher AUC of model 2 implied that supplemented probiotics (*Akkermansia muciniphila*, *Bacteroides eggerthii*, and *Butyricimonas synergistic*) may improve bone metabolism, which needs to be verified as a treatment effect on OP while avoiding drug-related side effects. All the above mechanistic evidence illustrates a significant and potential role in OP, which may provide a new direction for the treatment of OP.

## Data Availability

The datasets presented in this study can be found in online repositories. The names of the repository/repositories and accession number(s) can be found below: https://www.ebi.ac.uk/ena, PRJEB36271.
