# Supplementary figures and images for: The Relationship Between Osteoporosis and Intestinal Microbes in the Henan Province of China
Source: Front Cell Dev Biol. 2021 Nov 18;9:752990. doi: 10.3389/fcell.2021.752990 (PMC8638085; doi:10.3389/fcell.2021.752990)

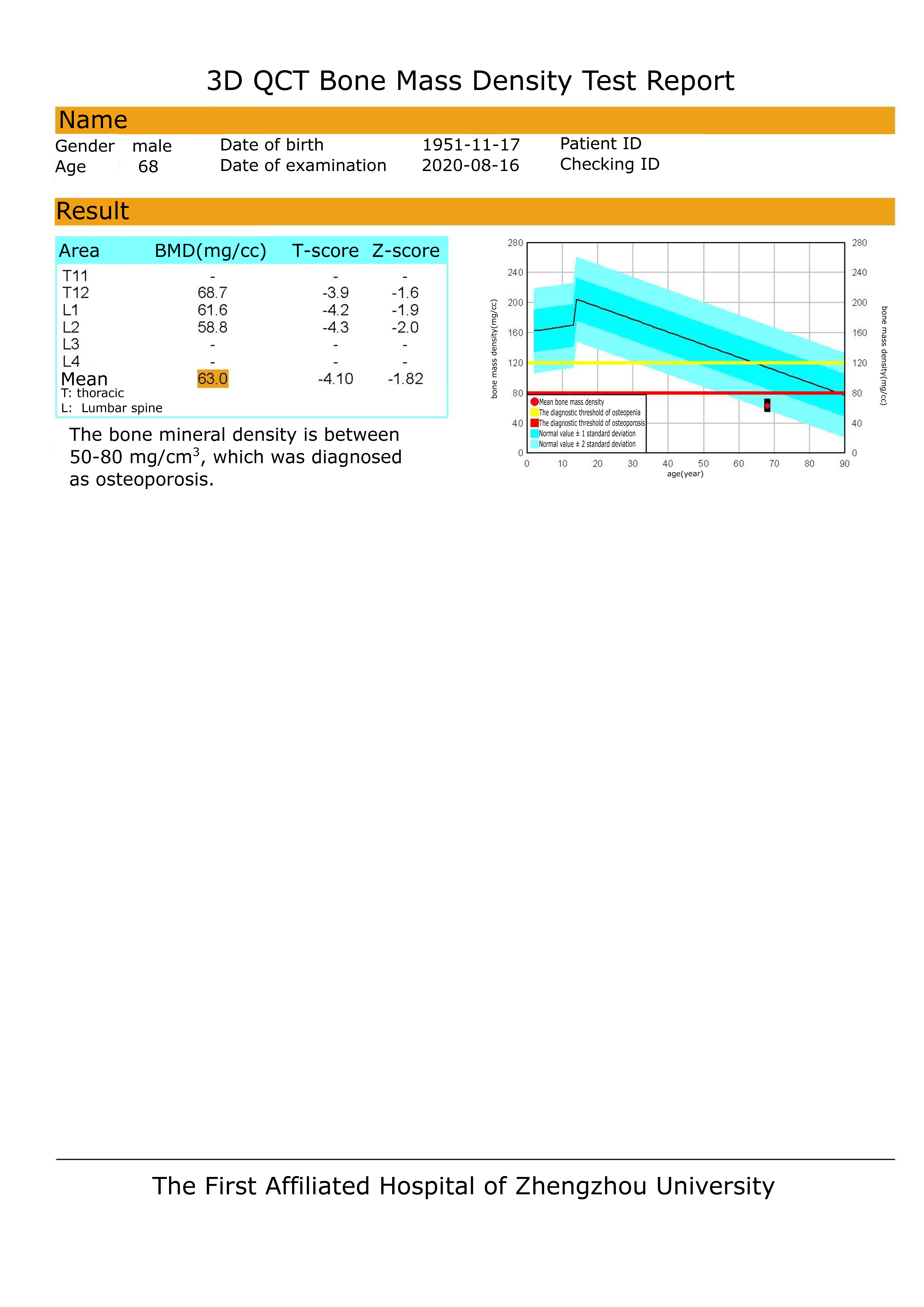

Supplement: Supplementary file 2 [file Image1.JPEG]

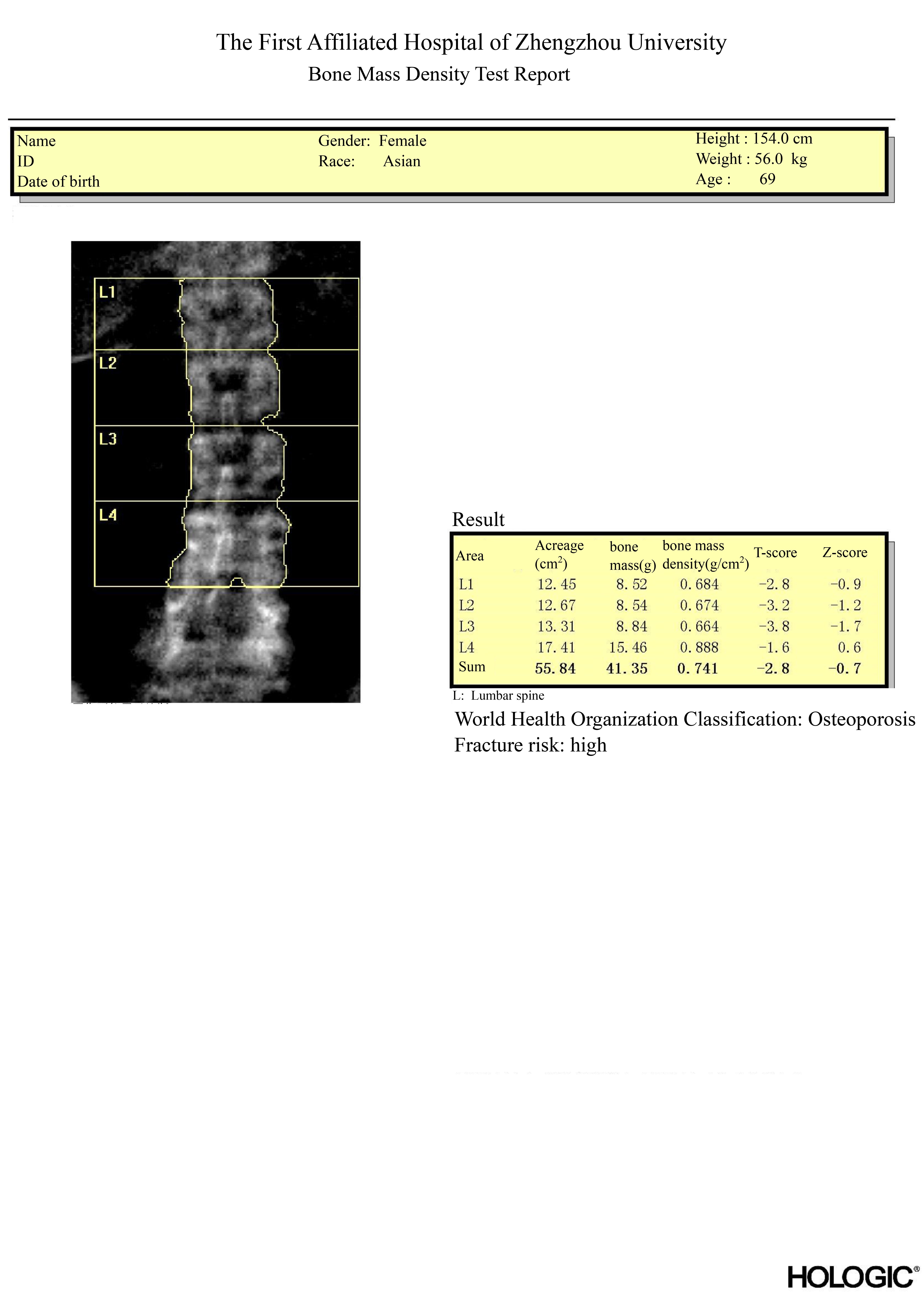

Supplement: Supplementary file 3 [file Image2.JPEG]
